# Advancing life: innovative approaches to enhance survival in sickle cell anemia patients

**DOI:** 10.1097/MS9.0000000000002534

**Published:** 2024-09-04

**Authors:** Emmanuel Ifeanyi Obeagu, Teddy Charles Adias, Getrude Uzoma Obeagu

**Affiliations:** a Department of Medical Laboratory Science, Kampala International University; b School of Nursing Science, Kampala International University, Kampala, Uganda; c Department of Haematology and Blood Transfusion Science, Faculty of Medical Laboratory Science, Federal University Otuoke, Bayelsa State, Nigeria

**Keywords:** blood transfusions, genetic therapies, medical interventions, mortality rates, sickle cell anemia

## Abstract

Sickle cell anemia (SCA) is a severe genetic disorder characterized by the production of abnormal hemoglobin S, leading to the formation of sickle-shaped red blood cells that cause chronic anemia, pain, and organ damage. This review explores recent innovative strategies aimed at improving survival rates and quality of life for SCA patients. Genetic therapies, particularly gene editing with CRISPR-Cas9 and gene therapy using lentiviral vectors, have shown significant potential in correcting the genetic defects responsible for SCA. Clinical trials demonstrate that these approaches can reduce sickle cell crises and minimize the need for blood transfusions by enabling the production of healthy red blood cells. Novel pharmacological treatments such as voxelotor, crizanlizumab, and L-glutamine provide additional mechanisms to prevent hemoglobin polymerization, reduce vaso-occlusive episodes, and decrease oxidative stress, respectively. These therapies offer new hope for patients, particularly those who do not respond adequately to existing treatments. Improved blood transfusion protocols, including automated red cell exchange and advanced donor-matching techniques, have enhanced the safety and efficacy of transfusions, reducing complications like alloimmunization. Comprehensive care models, integrating multidisciplinary care teams, patient education, and telemedicine, have further contributed to better disease management. By providing holistic care that addresses both medical and psychosocial needs, these models improve patient adherence to treatment and overall health outcomes. This review highlights the importance of these innovative strategies and calls for continued research and development to sustain and expand these advancements in SCA care.

## Introduction

Highlights

- Genetic therapies: Advancements in CRISPR-Cas9 and lentiviral gene therapy show promise in correcting the genetic defect causing SCA.
- Novel pharmacological agents: Drugs like voxelotor, crizanlizumab, and L-glutamine reduce SCA complications.
- Improved blood transfusion protocols: Automated red cell exchange and advanced donor matching enhance transfusion safety and efficacy.
- Comprehensive care models: Multidisciplinary teams, patient education, and telemedicine provide holistic, continuous care, addressing medical and psychosocial needs.
- Global health initiatives: Strengthening healthcare infrastructure and international collaboration ensures advancements benefit SCA patients globally, especially in low-resource settings.

Sickle cell disease is a genetic disorder affecting the red blood cells in the body. Normally, red blood cells are round and flexible, allowing them to move easily through blood vessels, but in individuals with sickle cell disease, the red blood cells become rigid and take on a crescent or sickle shape. These abnormally shaped cells can get stuck in small blood vessels, leading to blockages that cause pain, organ damage, and a range of other complications^1,2^. SCD is caused by a mutation in the gene that instructs the body to make hemoglobin, the molecule in red blood cells that carries oxygen. This mutation causes the production of abnormal hemoglobin known as hemoglobin S (HbS). When oxygen levels are low or when the blood cells are exposed to stress, the HbS molecules can cause the red blood cells to become stiff and form the characteristic sickle shape^3,4^. There are different types of sickle cell disease, with the most common being sickle cell anemia (SS), where an individual inherits two copies of the abnormal gene (one from each parent). Other forms include hemoglobin SC disease (HbSC) and sickle beta-thalassemia (SBthal), where an individual inherits one copy of the abnormal gene along with another abnormal gene or a gene mutation^5,6^.

SCD can lead to various health problems such as anemia, increased susceptibility to infections, organ damage, stroke, acute pain crises known as sickle cell crises, and other complications. Management and treatment strategies include medications, blood transfusions, hydroxyurea, pain management, and sometimes bone marrow or stem cell transplants aimed at alleviating symptoms and reducing complications^7^. It’s a condition that requires ongoing medical care and attention, and research into new treatments and potential cures is ongoing. Efforts to raise awareness, improve access to care, and provide support to individuals and families affected by SCD are essential in managing this condition effectively. Managing sickle cell disease (SCD) poses several significant challenges due to its complex nature and the wide range of potential complications associated with the condition. Sickle cell crises can cause severe and unpredictable pain episodes. These episodes often require immediate medical attention and pain management, which may include medications, hydration, and sometimes hospitalization^8^. SCD can affect various organs and systems in the body, leading to complications such as stroke, acute chest syndrome, pulmonary hypertension, kidney damage, vision problems, and more. Treating and preventing these complications require a multidisciplinary approach involving specialists in different medical fields^9^. Historically, treatment options for SCD have been limited. While certain medications like hydroxyurea can help reduce the frequency of pain crises, not all patients respond to these treatments, and they might not be suitable for everyone^8^. Access to specialized care for individuals with SCD can be challenging, especially in areas with limited healthcare resources or in communities with disparities in healthcare access. This lack of access can lead to inadequate management and increased complications^9^. SCD can significantly impact a person’s quality of life. Chronic pain, frequent hospitalizations, and potential disabilities can lead to emotional stress, depression, and anxiety. Patients often require mental health support along with medical care. As individuals with SCD transition from pediatric to adult healthcare settings, they might face difficulties in maintaining consistent and comprehensive care. This transition can lead to gaps in care and challenges in managing the disease effectively. There’s a need for continued research and the development of new therapies, including potential gene-based treatments or stem cell transplantation, to offer more effective and curative options for those living with SCD^10^. Lack of awareness among both healthcare providers and the general population about SCD can result in delayed diagnosis, inadequate care, and stigmatization of individuals affected by the disease^11^. Addressing these challenges requires a holistic approach involving healthcare providers, researchers, policymakers, and community organizations to improve access to care, develop better treatment options, raise awareness, and provide support for individuals and families affected by SCD.

SCD can lead to a high mortality rate due to various factors associated with the condition. SCD can cause damage to various organs in the body, including the lungs, kidneys, liver, and brain. Over time, this damage can become severe and lead to organ failure, contributing to a higher mortality rate^12^. Individuals with SCD have weakened immune systems, making them more susceptible to infections. Certain infections, particularly in those with compromised immune systems, can be severe and life-threatening. SCD is characterized by episodes of acute pain known as sickle cell crises, which can be extremely severe and require immediate medical attention. If not managed promptly and effectively, these crises can lead to serious complications or even death. People with SCD are at a higher risk of experiencing a stroke, particularly at a younger age. Strokes can cause severe disabilities or can be fatal^10^. Historically, treatment options for SCD have been limited. While certain treatments, such as hydroxyurea, can help manage the disease, not all patients respond well to these therapies. Additionally, access to specialized care and treatments can be limited in certain regions or communities, leading to inadequate management of the disease^13^. Disparities in healthcare access, socioeconomic factors, and healthcare provider awareness can lead to delays in diagnosis, inadequate care, and poorer health outcomes for individuals with SCD, contributing to a higher mortality rate. The chronic pain, frequent hospitalizations, and potential disabilities associated with SCD can lead to emotional stress, mental health issues, and poor quality of life, which might indirectly contribute to a higher mortality rate^14^. Reducing the mortality rate associated with SCD requires a multifaceted approach that involves improving access to comprehensive care, enhancing awareness among healthcare providers and the public, developing better treatment options, providing adequate support for mental health, and addressing healthcare disparities to ensure timely and effective management of the disease. Advances in research, increased education, and better access to specialized care can significantly impact the mortality rate for individuals living with SCD. Table 1 shows summary of the Review (provided by the authors).

**Table 1:** Summary of the review

Section	Summary
Abstract	Highlights the impact of sickle cell anemia (SCA) on survival rates and introduces innovative strategies to improve outcomes, including gene therapy and novel treatments
Keywords	Sickle cell anemia; Mortality rates; Medical interventions; Blood transfusions; Genetic therapies
Introduction	Discusses SCA as a genetic disorder with significant morbidity and mortality, emphasizing the need for innovative approaches to enhance survival rates
Innovative strategies	Summarizes key innovative approaches:
	− Gene therapy: CRISPR/Cas9 techniques to correct genetic mutations or increase fetal hemoglobin levels
	− Novel pharmacological agents: New drugs like voxelotor and crizanlizumab that target sickle cell pathology
	− Comprehensive care models: Multidisciplinary approaches improving patient management and outcomes
	− Advancements in supportive therapies: Enhanced pain management and transfusion practices to reduce complications
Gene therapy: CRISPR/Cas9	Describes CRISPR/Cas9 technology for targeted gene correction and fetal hemoglobin induction, supported by preclinical and clinical trial data
Gene addition techniques	Discusses lentiviral vectors for gene addition, showcasing empirical data from clinical trials indicating improved hemoglobin levels and reduced pain crises
Conclusion	Emphasizes the importance of innovative strategies in transforming care for SCA patients and the need for ongoing research and collaboration in the field
References	Lists studies and articles supporting the review’s findings, underscoring the importance of empirical data in validating innovative approaches

## Aim

The primary aim of this review article is to explore and elucidate innovative strategies that have demonstrated potential in improving the survival rates and quality of life for patients with sickle cell anemia (SCA).

## Rationale

SCA is a debilitating genetic disorder that imposes significant morbidity and mortality burdens on affected individuals, particularly in regions with limited healthcare resources. Despite advancements in medical research and treatment, SCA continues to present substantial challenges in its management due to its complex pathophysiology and the variability of its clinical manifestations. The need for innovative and effective treatment strategies is paramount to improve survival rates and the quality of life for SCA patients. Traditional treatments for SCA, such as hydroxyurea and regular blood transfusions, primarily focus on managing symptoms rather than addressing the underlying genetic defect. Genetic therapies, including gene editing and gene therapy, offer the potential to correct the root cause of SCA at the molecular level. The rapid advancements in technologies like CRISPR-Cas9 and lentiviral vectors have opened new avenues for potentially curative treatments. By exploring these cutting-edge approaches, this review aims to highlight the transformative potential of genetic therapies in SCA management. The introduction of novel pharmacological agents such as voxelotor, crizanlizumab, and L-glutamine has broadened the therapeutic landscape for SCA. These medications target different aspects of the disease’s pathophysiology, offering new mechanisms of action that can complement existing treatments. Reviewing these pharmacological advancements provides insights into how these drugs can reduce complications, enhance patient outcomes, and offer alternative options for those who do not respond adequately to traditional therapies.

Blood transfusions remain a cornerstone in the management of severe SCA complications. However, the risks associated with alloimmunization and iron overload necessitate improved transfusion protocols. Innovations such as automated red cell exchange and extended phenotype matching have significantly enhanced the safety and efficacy of transfusions. Evaluating these protocols underscores their importance in reducing adverse effects and improving the long-term health of SCA patients. Effective management of SCA requires a holistic approach that addresses both medical and psychosocial aspects of the disease. Multidisciplinary care teams, patient education, self-management strategies, and telemedicine are crucial components of comprehensive care models that can significantly enhance patient outcomes. By assessing these integrated care approaches, this review aims to highlight the importance of a coordinated and patient-centered strategy in managing SCA. Despite the progress made in SCA treatment, there remain gaps in research and clinical practice that need to be addressed. This review aims to identify these gaps and suggest future directions for research and healthcare policy. By doing so, it seeks to provide a roadmap for continued advancements and improvements in the management of SCA.

## Review methodology

The review methodology outlines the systematic approach used to gather, evaluate, and synthesize information on innovative strategies to improve survival rates in SCA patients. This methodology ensures the comprehensiveness and reliability of the findings presented in the review.

## Literature search

### Databases

A comprehensive literature search was conducted using the following electronic databases:

- PubMed
- MEDLINE
- Scopus
- Web of Science
- Google Scholar

### Search terms

The search strategy incorporated a combination of keywords and medical subject headings (MeSH) to ensure thorough coverage. Key search terms included:

- “Sickle cell anemia”
- “Sickle cell disease”
- “Gene therapy”
- “Gene editing”
- “CRISPR-Cas9”
- “Voxelotor”
- “Crizanlizumab”
- “L-glutamine”
- “Blood transfusion protocols”
- “Automated red cell exchange”
- “Comprehensive care”
- “Multidisciplinary care”
- “Telemedicine”

### Inclusion and exclusion criteria

To ensure relevance and quality, the following inclusion and exclusion criteria were applied:

#### Inclusion criteria

- Peer-reviewed articles
- Studies and reviews focusing on innovative treatments and management strategies for SCA
- Clinical trials, meta-analyses, and systematic reviews
- Articles in English

#### Exclusion criteria

- Non-peer-reviewed articles
- Case reports and opinion pieces
- Articles not focused on SCA

### Early diagnosis and intervention of sickle cell anemia

Early diagnosis and intervention are pivotal in managing this condition effectively^15–17^. Identifying the condition in infancy or early childhood allows for prompt medical intervention and the initiation of appropriate therapies to prevent complications^18^. Early diagnosis enables healthcare providers to implement preventive measures such as penicillin prophylaxis and vaccinations against infections like pneumococcal disease, reducing the risk of life-threatening illnesses^19^. Early diagnosis provides families with the opportunity to receive education and counseling about the condition, its management, and the potential challenges, empowering them to better care for their child^20^.

Many countries have established universal newborn screening programs to identify infants with sickle cell disease shortly after birth. This simple blood test detects the presence of abnormal hemoglobin and is a highly effective method for early diagnosis^21^. Genetic counseling and carrier testing are valuable tools for identifying individuals with sickle cell trait. Couples planning to start a family can be tested to assess their risk of having a child with SCA. This information allows for informed family planning decisions and early prenatal care if needed^22^. Healthcare providers, caregivers, and teachers should be educated about the early signs and symptoms of SCA, such as pain crises, fatigue, jaundice, and pallor. Timely recognition of these symptoms can lead to early medical evaluation and intervention^23^. Genetic counseling services can assist families in understanding the genetic basis of SCA, assess the risk of the disease in future pregnancies, and discuss available options for family planning^24^. In cases where a family has a history of SCA or both parents are carriers, prenatal testing can be conducted to diagnose the condition before birth. This allows for early planning and preparation for the care of the affected child^25^. Once diagnosed, SCA patients should receive comprehensive care from a team of healthcare professionals, including hematologists, pediatricians, and nurses, to manage the condition and its complications effectively^26^. Early diagnosis and intervention of sickle cell anemia are critical for improving the quality of life and long-term outcomes for affected individuals. Through universal newborn screening, genetic counseling, and timely medical management, healthcare providers and families can work together to ensure that those with SCA receive the care and support they need from the earliest stages of life^27^.

### Hydroxyurea therapy in SCA

Hydroxyurea, a medication first introduced as an anticancer agent, has proven to be a significant advancement in the treatment of SCA. SCA is a hereditary blood disorder characterized by the production of abnormal hemoglobin, leading to the deformation of red blood cells and a wide range of health complications. This article focuses on the use of hydroxyurea therapy in the management of SCA, its mechanisms of action, benefits, and considerations^28–32^. Hydroxyurea stimulates the production of fetal hemoglobin, a type of hemoglobin unaffected by the sickle cell mutation. Elevated levels of HbF reduce the proportion of abnormal hemoglobin (HbS) in the bloodstream, leading to a decrease in the formation of sickled red blood cells^33^. Hydroxyurea therapy results in fewer sickle cells, which leads to reduced hemolysis. This, in turn, lessens the release of free hemoglobin into the bloodstream, decreasing oxidative stress and inflammation^34^. Hydroxyurea increases the release of nitric oxide in the blood vessels, promoting vasodilation and improved blood flow. This helps prevent vaso-occlusive crises and acute chest syndrome^35^. Hydroxyurea significantly reduces the frequency and severity of pain crises in individuals with SCA, improving the overall quality of life for patients. This reduction in pain crises results in fewer hospitalizations and a decreased need for strong pain medications^36^. Acute chest syndrome is a life-threatening complication of SCA. Hydroxyurea therapy lowers the risk of developing this condition, which is characterized by lung inflammation and is often triggered by infections^37^. With fewer vaso-occlusive crises and complications, patients on hydroxyurea experience fewer hospitalizations, reducing the burden on healthcare systems and improving patients’ overall well-being^38^. Hydroxyurea can help children with SCA grow and develop more normally by reducing the impact of chronic anemia on their bodies^39^.

The use of hydroxyurea should be tailored to each patient’s needs, and the dosage may need to be adjusted over time. Regular medical follow-ups are essential to monitor treatment effectiveness and possible side effects^40^. Patients on hydroxyurea therapy should be carefully monitored for potential side effects, such as myelosuppression (reduced blood cell production) or liver and kidney function abnormalities^41^. Women of childbearing age should use contraception while on hydroxyurea, as the drug may cause birth defects^42^. Patients and their families should receive thorough education about the medication, its effects, and the importance of adherence to the treatment plan^43^. Hydroxyurea therapy has emerged as a valuable tool in the management of sickle cell anemia. By increasing fetal hemoglobin levels and improving various aspects of the disease’s pathophysiology, it reduces pain crises, decreases the risk of acute chest syndrome, and minimizes hospitalizations. However, its use must be individualized and closely monitored to ensure its safety and efficacy. Ongoing research in this field continues to enhance our understanding of hydroxyurea therapy and may lead to further refinements in the treatment of SCA^44^.

Hydroxyurea (HU) therapy is one of the primary treatments used for individuals with SCD. It’s an oral medication that has shown effectiveness in reducing the frequency and severity of pain crises and other complications associated with SCD. One of the most significant concerns with HU is its potential to suppress bone marrow function, leading to decreased production of red blood cells, white blood cells, and platelets. This suppression can cause anemia, leukopenia (low white blood cell count), and thrombocytopenia (low platelet count)^40^. Due to the suppression of white blood cells, individuals taking HU may be more susceptible to infections. Lowered immunity can increase the risk and severity of infections, which can be particularly concerning in populations already prone to infections due to SCD-related immune system issues^41^. There are concerns about the impact of HU on fertility and reproductive health. Some studies have suggested potential risks to fertility in both men and women, as well as potential risks to developing fetuses during pregnancy^40^. While short-term safety has been established in many studies, the long-term effects of taking HU for extended periods are not fully understood. Continuous monitoring is necessary to assess any potential risks associated with prolonged use. Not all individuals with SCD respond the same way to HU therapy. Some may experience significant benefits, while others may not show improvements or may experience adverse effects that outweigh the benefits^42^. HU requires consistent and regular intake for its effectiveness. Patient adherence to the prescribed regimen might be challenging due to factors such as pill burden, side effects, or other personal reasons^41^. There might be concerns about using HU in certain populations, such as pediatric patients, pregnant women, or individuals with kidney or liver impairment. Dosage adjustments and careful monitoring are necessary in these cases^40^. Addressing these challenges involves careful monitoring of patients on HU therapy, regular blood tests to assess blood counts and organ function, and close collaboration between healthcare providers and patients to manage potential side effects. Additionally, ongoing research aims to develop alternative treatments or combination therapies that can provide benefits while minimizing the limitations associated with HU. Personalized medicine approaches may also help identify patients who are most likely to benefit from HU therapy while minimizing risks.

### Blood transfusions in SCA

Blood transfusions have proven to be a vital therapeutic intervention in the management of SCA, offering relief from symptoms and preventing life-threatening complications. This article focuses on the use of blood transfusions in the treatment of SCA, their mechanisms of action, benefits, and considerations^45–47^. SCA patients often have lower hemoglobin levels due to chronic hemolysis and poor production of normal hemoglobin. Transfusions provide a quick way to increase hemoglobin levels, improving the oxygen-carrying capacity of the blood^48^. Transfused red blood cells are typically normal in shape and function, diluting the proportion of sickled red blood cells in the patient’s circulation. This helps reduce vaso-occlusive crises and other complications associated with the aggregation of sickled cells^49^. The transfusion of healthy red blood cells enhances the delivery of oxygen to tissues and organs, alleviating symptoms of anemia and reducing the risk of complications such as acute chest syndrome^50^. Blood transfusions can provide rapid relief from vaso-occlusive pain crises, which are characterized by severe pain and tissue damage due to blocked blood vessels. Transfusions help alleviate pain by improving blood flow^51^. Children with SCA are at risk of stroke due to cerebral blood vessel occlusion. Chronic blood transfusions may be used to reduce this risk significantly^52^. Regular transfusions can lower the likelihood of acute chest syndrome, a life-threatening condition characterized by lung inflammation and pulmonary complications^53^. Blood transfusions can lead to an overall improvement in a patient’s quality of life, reducing the frequency of hospitalizations and the need for strong pain medications^54^.

Blood transfusions must be matched carefully to ensure compatibility between the donor’s blood type and the recipients. Cross-matching is performed to reduce the risk of adverse reactions^55^. Frequent blood transfusions can lead to iron overload in the body, which can cause organ damage. Patients may require iron-chelation therapy to manage excess iron^55^. While efforts are made to match blood types accurately, there is still a risk of transfusion reactions, which can range from mild to severe. Monitoring for reactions is essential during and after transfusions^55^. Blood transfusions carry a small risk of transmitting infectious diseases. Rigorous screening and testing of donated blood help minimize this risk^55^. Blood transfusions have become a critical component of the comprehensive management of SCA. By increasing hemoglobin levels, diluting the proportion of sickled red blood cells, and improving oxygen delivery to tissues and organs, transfusions provide relief from symptoms, reduce the frequency of complications, and enhance the overall quality of life for individuals with SCA. However, careful monitoring and management of iron overload and potential transfusion reactions are essential to ensure the safety and efficacy of this therapy. Collaborative efforts among healthcare providers and patients are crucial to achieving optimal outcomes in the treatment of SCA through blood transfusions^56^.

### Bone marrow transplantation in SCA

While advances in medical treatments have improved the quality of life for SCA patients, bone marrow transplantation has emerged as a potential curative option for those with severe forms of the disease. This paper discusses the use of bone marrow transplantation in the management of SCA, its mechanisms, benefits, and considerations^57^. Bone marrow transplantation, also known as hematopoietic stem cell transplantation, is a procedure that involves replacing the patient’s abnormal bone marrow with healthy donor marrow^58^. The primary mechanisms of action in bone marrow transplantation for SCA are as follows: In SCA, the bone marrow produces abnormal red blood cells due to a genetic mutation. A successful bone marrow transplant introduces healthy hematopoietic stem cells that produce normal hemoglobin, thereby preventing the production of sickled red blood cells^59^. A successful bone marrow transplant can result in long-term correction of the underlying genetic defect. This not only alleviates the symptoms of SCA but can potentially cure the disease^59^.

For individuals with severe forms of SCA, bone marrow transplantation can offer the possibility of a cure, as it corrects the genetic defect responsible for the disease^60^. Successful transplantation results in the production of healthy red blood cells, effectively eliminating the symptoms and complications associated with SCA^60^. By providing a continuous source of normal red blood cells, bone marrow transplantation can prevent organ damage, including damage to the heart, lungs, and kidneys, which can occur in untreated SCA^60^. Patients who undergo successful transplantation can experience a significant improvement in their overall quality of life, as they are no longer burdened by the chronic pain, anemia, and frequent hospitalizations associated with SCA^60^. Finding a suitable donor with a closely matched tissue type is crucial for the success of the procedure. Family members are often preferred donors, but unrelated donors may also be considered^61^. After transplantation, there is a risk that the donor’s immune cells may attack the recipient’s tissues, a condition known as graft-versus-host disease (GVHD). Medications and careful management are necessary to mitigate this risk^61^. Prior to transplantation, patients typically undergo conditioning therapy, which involves chemotherapy and sometimes radiation to suppress the recipient’s immune system and create space in the bone marrow for the transplanted cells^61^. Patients who undergo bone marrow transplantation require lifelong monitoring to ensure the success of the procedure and to address potential complications^61^. Bone marrow transplantation represents a potential cure for severe Sickle Cell Anemia and offers significant relief from the debilitating symptoms and complications associated with the disease. While it is a complex and invasive procedure with potential risks, the advancements in transplantation techniques, donor matching, and post-transplant care have improved the chances of success. Patients and their healthcare providers must carefully consider the benefits and risks of bone marrow transplantation in the context of their specific SCA condition to determine if it is a suitable treatment option^62^.

### Treatment of acute complications

In SCA, acute complications can arise due to the abnormal sickle-shaped red blood cells blocking blood vessels, leading to tissue damage and reduced oxygen supply. Managing these acute complications involves addressing symptoms promptly to prevent further complications and improve patient outcomes^2^. Pain crises are often managed with analgesic medications to relieve pain. Nonsteroidal anti-inflammatory drugs (NSAIDs), opioids, and other pain medications are used to control pain severity^63^. Adequate hydration is essential to prevent further sickling of red blood cells. Intravenous fluids are often administered to maintain hydration and improve blood flow. Providing oxygen therapy helps increase the oxygen level in the blood, reducing the severity of pain and tissue damage. Similar to pain crises, supplemental oxygen is provided to maintain adequate oxygen levels and alleviate respiratory distress. Broad-spectrum antibiotics are commonly used to treat infections that may contribute to acute chest syndrome. In severe cases, blood transfusions may be required to improve oxygenation and replace damaged red blood cells. In cases of severe splenic sequestration (sudden trapping of blood in the spleen), immediate blood transfusions are often necessary to stabilize the patient and prevent further complications^64^. For patients at high risk of stroke or those who have had a stroke, regular blood transfusions are used to lower the percentage of sickle hemoglobin (HbS) and reduce the risk of recurrent strokes^65^. Similar to other acute complications, maintaining hydration and managing pain are essential. Analgesics and hydration help reduce the duration and severity of priapism. In cases of severe acute anemia due to SCA complications, blood transfusions may be required to rapidly increase the number of healthy red blood cells and improve oxygen delivery. It’s important to note that treatment plans for acute complications in SCA are tailored to each individual based on the severity of symptoms, patient history, and other medical considerations. Close monitoring, prompt intervention, and multidisciplinary care involving hematologists, pain specialists, and other healthcare professionals are essential in managing acute complications effectively and minimizing long-term complications.

### Prevention of stroke

In individuals with SCA, prevention of stroke is a crucial aspect of managing the disease, particularly in children who are at higher risk. Stroke prevention strategies focus on reducing the risk of cerebrovascular complications associated with sickle cell disease^65^. Regular TCD ultrasound screening is recommended for children with SCA to assess their risk of stroke^66^. TCD measures blood flow velocity in the brain’s blood vessels. High blood flow velocities are associated with an increased risk of stroke. Children identified as high risk through TCD screening may require additional interventions to prevent stroke. Children identified as high risk for stroke based on TCD screening may undergo chronic blood transfusions to reduce the risk. Regular transfusions help lower the percentage of sickle hemoglobin (HbS) and decrease the likelihood of red blood cells sticking together and blocking blood vessels in the brain^66^. Hydroxyurea, an oral medication, has been shown to decrease the frequency and severity of pain crises and complications in SCA. It might also help reduce the risk of stroke by increasing fetal hemoglobin levels and improving blood flow^67^. Educating patients, caregivers, and healthcare providers about the signs and symptoms of stroke is essential. Early recognition of stroke symptoms such as weakness, speech difficulty, and sudden changes in vision can lead to prompt medical intervention, reducing the potential impact of a stroke^65^. For individuals identified as high risk based on TCD screening, close monitoring and adherence to the recommended treatment plan are critical. Regular follow-ups with a hematologist or a specialist experienced in SCA management are necessary to ensure appropriate management and prevent stroke occurrence^66^. Managing other risk factors that might contribute to stroke, such as hypertension, diabetes, and high cholesterol, is important in reducing the overall risk of stroke in individuals with SCA^65^. Children with SCA are at increased risk of bacterial infections, including those that could lead to a stroke^65^. Vaccinations (e.g. pneumococcal vaccines) and prophylactic antibiotics might be recommended to prevent infections that could potentially lead to cerebrovascular complications. The prevention of stroke in SCA requires a multifaceted approach, including regular monitoring, individualized treatment plans, patient education, and close collaboration between patients, caregivers, and healthcare providers. Identifying high-risk individuals and implementing appropriate interventions can significantly reduce the risk of stroke and its potential impact on individuals with Sickle Cell Anemia.

### Chronic transfusions

Chronic transfusions are a treatment option used in individuals with severe SCA to manage complications and reduce the risk of certain complications associated with the disease. This treatment involves regularly scheduled blood transfusions to help prevent or alleviate complications related to sickle cell disease^63^. Chronic transfusions are typically considered for individuals with SCA who have had complications such as recurrent strokes, severe anemia, acute chest syndrome, or other severe complications. Additionally, children identified as high risk for stroke based on transcranial Doppler (TCD) screening might be candidates for chronic transfusions to prevent stroke occurrence^65^. The primary goal of chronic transfusions in SCA is to reduce the percentage of sickle hemoglobin (HbS) in the blood and increase the levels of healthy red blood cells. By increasing the proportion of normal red blood cells, chronic transfusions can help prevent the formation of sickle-shaped cells, reduce the risk of complications such as stroke, and improve overall well-being^2^. Chronic transfusions are typically administered on a regular schedule, often every 3–4 weeks. The frequency and duration of transfusions may vary depending on the individual’s medical history, response to treatment, and the specific goals of therapy^62^. While chronic transfusions can be beneficial, they come with potential risks and challenges. These include the risk of iron overload due to repeated transfusions, which can lead to complications in various organs such as the heart, liver, and endocrine system. Iron-chelation therapy might be necessary to manage iron overload in individuals undergoing chronic transfusions^64^. There is also a risk of developing antibodies against foreign blood cells (alloimmunization) with repeated transfusions, which can complicate future transfusions. Additionally, infections, transfusion reactions, and other adverse effects are potential risks associated with chronic transfusion therapy. Individuals undergoing chronic transfusions require close monitoring by healthcare providers. Regular blood tests, including iron levels and organ function tests, are performed to assess the impact of transfusion therapy and manage potential complications. For some patients, other therapies such as hydroxyurea or hematopoietic stem cell transplantation might be considered as alternatives or adjuncts to chronic transfusion therapy, depending on individual circumstances and treatment goals. The decision to initiate chronic transfusion therapy in individuals with SCA is based on careful consideration of the risks and benefits, the individual’s medical history, the presence of complications, and the goals of treatment. Close collaboration between hematologists, transfusion specialists, and other healthcare providers is essential in managing chronic transfusion therapy effectively while minimizing risks and optimizing outcomes for patients with severe SCA.

### Iron overload

Iron overload is a condition characterized by an excess accumulation of iron in the body, which can lead to various health complications. In the context of SCA, iron overload often occurs as a result of chronic blood transfusions used to manage complications of the disease^68^. Chronic blood transfusions, a common treatment for SCA, introduce additional iron into the body. Each unit of transfused blood contains iron, and frequent transfusions can lead to an accumulation of iron over time. In individuals with SCA who require regular transfusions, the body’s natural mechanisms for regulating iron levels might become overwhelmed, leading to iron overload^68^. Excess iron accumulation can result in damage to various organs, including the heart, liver, pancreas, and endocrine glands. Excessive iron deposition in the heart can lead to heart problems, including cardiomyopathy (weakening of the heart muscle) and heart failure. Iron overload can cause liver damage, leading to conditions such as fibrosis, cirrhosis, and liver failure. Iron overload can affect the endocrine system, leading to hormonal imbalances and issues such as diabetes, hypothyroidism, and delayed puberty. Excess iron can accumulate in other organs, causing damage and dysfunction in the pancreas, joints, and other tissues^69^. Regular monitoring of iron levels through blood tests, such as serum ferritin levels, helps assess the extent of iron overload. Imaging studies like MRI (magnetic resonance imaging) can also measure iron levels in specific organs. Treatment for iron overload often involves iron-chelation therapy. Chelation agents (e.g. deferoxamine, deferiprone, deferasirox) are medications that bind to excess iron and help remove it from the body through urine or stool. Strategies to prevent iron overload in individuals receiving chronic transfusions include careful monitoring of iron levels, adjusting transfusion schedules when appropriate, and starting iron chelation therapy early to prevent excessive iron accumulation. Patients and caregivers should be educated about the importance of adherence to treatment plans, including regular blood tests and iron chelation therapy, to prevent or manage iron overload effectively. Managing iron overload in individuals with SCA who require chronic transfusions is crucial to prevent complications and maintain overall health. Close monitoring, early intervention, and appropriate management strategies, including iron chelation therapy, are essential components of care for those at risk of or experiencing iron overload.

### Alloimmunization

Alloimmunization refers to the development of antibodies against foreign blood cells, particularly red blood cell antigens, as a result of exposure to non-self-antigens through blood transfusions or during pregnancy. In the context of SCA and chronic blood transfusions, alloimmunization poses a significant concern and can lead to complications during subsequent transfusions^70^. Individuals receiving frequent blood transfusions, such as those with SCA who require chronic transfusion therapy, are at risk of developing alloantibodies against foreign blood cell antigens. These antibodies are formed when the recipient’s immune system recognizes antigens on donor red blood cells as foreign and mounts an immune response against them. Alloimmunization can result in the formation of antibodies that target specific red blood cell antigens. Once formed, these antibodies can cause adverse reactions during subsequent transfusions, including hemolytic transfusion reactions, where the recipient’s immune system attacks and destroys transfused red blood cells. This can lead to transfusion reactions, anemia, and other complications^70^. Alloantibodies can complicate future transfusions by causing difficulties in finding compatible blood for transfusion, known as cross-matching difficulties. This can limit the available blood products for transfusion and increase the risk of delayed or ineffective transfusion, potentially leading to complications in managing SCA-related conditions. Strategies to prevent alloimmunization include providing matched blood transfusions whenever possible, reducing the number of transfusions when clinically appropriate, and minimizing exposure to different blood antigens. For individuals who have already developed alloantibodies, identified compatible blood products or used special blood processing techniques to remove antibodies may be necessary. Regular screening and monitoring for the presence of antibodies in individuals receiving chronic transfusions are essential. Testing involves screening for unexpected antibodies and identifying specific antibodies if present. This information helps guide future transfusion strategies to minimize transfusion reactions^70^. Patients with SCA and their caregivers should be educated about the risks of alloimmunization and the importance of regular follow-ups, antibody screening, and close monitoring to manage and prevent complications associated with transfusions. Alloimmunization remains a significant concern in individuals with SCA who require chronic transfusion therapy. Close monitoring, proper screening, and efforts to minimize exposure to different blood antigens are crucial in managing alloimmunization and ensuring safe and effective transfusion therapy for individuals with Sickle Cell Anemia.

### Infections

Infections are a significant concern for individuals with SCA due to the impact of the disease on the immune system and the increased susceptibility to certain infections^71^. SCA can compromise the immune system, making individuals more susceptible to bacterial infections, particularly encapsulated bacteria such as Streptococcus pneumoniae, Haemophilus influenzae type b, and Neisseria meningitidis^72^. Sickle cell disease can lead to functional asplenia, where the spleen becomes damaged or non-functional due to repeated sickling of red blood cells. As a result, individuals with SCA have reduced ability to fight off certain infections, particularly those caused by encapsulated bacteria, which are normally cleared by the spleen^71^. Chronic inflammation, a hallmark of SCA, can affect the immune response and make individuals more susceptible to infections^71^. SCA-related complications, such as damage to the lungs, kidneys, and other organs, can increase susceptibility to infections and make it harder to fight off pathogens. In addition to functional asplenia, some individuals with SCA may develop hyposplenism, where the spleen’s function is reduced but not completely lost. This condition also contributes to increased susceptibility to certain infections. Immunizations, including routine childhood vaccinations and additional vaccinations targeting encapsulated bacteria (e.g. pneumococcal, meningococcal, and Hib vaccines), are recommended to reduce the risk of infections. Long-term or periodic antibiotic prophylaxis might be prescribed to prevent certain infections, especially for young children or those at higher risk. Individuals with SCA and their caregivers should seek prompt medical attention for any signs or symptoms of infection to ensure timely treatment. Adequate hydration is essential to prevent the sickling of red blood cells, which can contribute to vaso-occlusive crises and increase the risk of infections. Managing infections in individuals with SCA involves a combination of preventive measures, vaccination, prompt medical care, and close monitoring to reduce the risk and severity of infections and their associated complications. To manage these risks, healthcare providers often monitor patients receiving frequent transfusions for signs of complications. Additionally, iron chelation therapy, which involves medications that help remove excess iron from the body, might be recommended to reduce iron overload in those who receive regular transfusions. The decision to administer blood transfusions in SCA is individualized based on the patient’s medical history, complications, and specific needs. The risks and benefits of transfusion therapy should be carefully evaluated and discussed between healthcare providers, patients, and their families.

### Gene therapy in SCA

Gene therapy for SCA involves the introduction of normal genetic material into the patient’s hematopoietic stem cells, with the primary aim of correcting the underlying genetic mutation responsible for the disease. Through various methods, gene therapy delivers functional copies of the beta-globin gene into the patient’s hematopoietic stem cells^73^. The introduction of corrected genes leads to increased production of normal hemoglobin (hemoglobin A) and a corresponding decrease in the proportion of abnormal hemoglobin (hemoglobin S). As a result, the patient’s red blood cells are less prone to sickling^73^. Gene therapy holds the potential to provide a long-term or permanent cure for SCA by addressing the root cause of the disease—the genetic mutation responsible for the production of abnormal hemoglobin^74^. Successful gene therapy results in the production of healthy red blood cells, effectively alleviating the painful symptoms and complications associated with SCA^74^. By providing a continuous source of normal red blood cells, gene therapy can prevent organ damage, such as damage to the heart, lungs, and kidneys, which can occur in untreated SCA^74^. Patients who undergo successful gene therapy can experience a significant improvement in their overall quality of life, as they are no longer burdened by chronic pain, anemia, and frequent hospitalizations^74^.

Gene therapy for SCA is an evolving field, and the safety and long-term efficacy of these treatments are still being studied. Clinical trials and research are ongoing to better understand the outcomes^75^. The choice of a delivery method for gene therapy is critical. It can involve the use of viral vectors or non-viral methods to introduce the corrected genes into the patient’s cells^75^. The body’s immune response to the introduced genetic material can impact the success of gene therapy. Immunomodulatory treatments may be necessary to manage any adverse reactions^75^. Patients who undergo gene therapy for SCA require lifelong monitoring to ensure the sustained effectiveness of the therapy and to address any potential complications or side effects^75^. Gene therapy represents a promising and innovative approach to treating SCA. By correcting the underlying genetic mutation responsible for the disease, gene therapy offers the potential for a long-term or permanent cure, effectively alleviating the symptoms and complications associated with SCA. As this field continues to evolve, patients and healthcare providers must carefully consider the benefits and risks of gene therapy in the context of the patient’s specific SCA condition and weigh these factors against other available treatment options. Continued research and clinical trials will further advance our understanding and application of gene therapy in the management of SCA^75^.

### Gene addition therapy

Gene addition therapy, also known as gene addition or gene addition/editing, is a therapeutic approach that involves introducing functional copies of genes into a patient’s cells to compensate for a defective or missing gene, ultimately correcting the underlying genetic condition. In the context of SCD, gene addition therapy aims to address the mutation in the HBB gene, responsible for producing abnormal hemoglobin (HbS)^74^. This technique involves introducing a functional copy of the beta-globin gene into the patient’s hematopoietic stem cells (HSCs), the precursor cells that give rise to all blood cells. Hematopoietic stem cells (HSCs) are collected from the patient’s bone marrow or peripheral blood through apheresis. These cells serve as the target for genetic modification. A functional copy of the beta-globin gene is introduced into the patient’s HSCs using viral vectors (such as lentiviruses or retroviruses). These vectors are modified to carry the correct beta-globin gene and are used as delivery vehicles to transport the corrected genetic material into the patient’s cells. The viral vectors containing the corrected beta-globin gene are introduced into the patient’s HSCs *ex vivo* (outside the body). The vectors are engineered to integrate the corrected gene into the DNA of the patient’s stem cells. The modified HSCs are cultured and expanded in the laboratory to increase their numbers. This step ensures that a sufficient number of genetically modified cells are available for transplantation back into the patient^75^. The genetically modified HSCs are then infused back into the patient’s bloodstream through a process similar to a stem cell transplant. Once inside the body, these modified stem cells will differentiate and produce healthy red blood cells with normal hemoglobin. Gene addition therapy aims to restore the production of normal hemoglobin, reducing the percentage of sickle hemoglobin (HbS) in the patient’s bloodstream. This approach holds promise as a potential curative treatment for sickle cell disease by addressing the root cause at the genetic level. Clinical trials and ongoing research are evaluating the safety, efficacy, and long-term outcomes of gene addition therapy for SCD. While this approach shows great potential, further studies are needed to optimize the technique, minimize potential risks, and ensure its safety and effectiveness before widespread clinical implementation.

### Gene editing (CRISPR/Cas9)

Gene editing using CRISPR/Cas9 technology is a cutting-edge approach that offers precision in modifying the genetic code, allowing targeted changes to be made in the DNA sequence^76^. In the context of SCD, CRISPR/Cas9 holds promise as a potential treatment by correcting the specific mutation in the HBB gene responsible for producing abnormal hemoglobin (HbS)^77^. Scientists identify the specific mutation in the HBB gene that leads to the production of abnormal hemoglobin responsible for SCD. CRISPR/Cas9 components consist of guide RNA (gRNA), which guides the Cas9 enzyme to the targeted location in the DNA sequence, and the Cas9 enzyme, which acts as molecular scissors to cut the DNA at the specific location of the mutation^76^. The designed CRISPR/Cas9 components are delivered into the patient’s hematopoietic stem cells (HSCs), which are the precursor cells for all blood cells. Once inside the cells, the guide RNA directs the Cas9 enzyme to the precise location of the HBB gene mutation, where the Cas9 enzyme induces a double-strand break in the DNA^76^. The cell’s natural repair mechanisms, such as non-homologous end joining (NHEJ) or homology-directed repair (HDR), can then repair the broken DNA. In the case of HDR, a corrected template with the normal sequence of the HBB gene can be provided to guide the repair process, resulting in the replacement of the mutated sequence with the corrected sequence^77^. The edited hematopoietic stem cells, now containing the corrected HBB gene sequence, undergo cell division and differentiation, giving rise to red blood cells with normal hemoglobin. CRISPR/Cas9 gene editing offers the potential for precise correction of the underlying genetic mutation responsible for SCD. By directly targeting and repairing the defective gene in the patient’s cells, this technology aims to restore the production of normal hemoglobin, reducing or eliminating the production of sickle hemoglobin (HbS)^76^. However, while CRISPR/Cas9 gene editing shows promising results in preclinical studies and research, challenges such as off-target effects, efficient delivery into target cells, long-term safety, and ethical considerations remain areas of active investigation. Ongoing clinical trials are being conducted to evaluate the safety, efficacy, and feasibility of using CRISPR/Cas9 technology for treating genetic disorders like Sickle Cell Disease.

### CRISPR and gene editing in SCA

Gene therapy, particularly through CRISPR and gene-editing techniques, represents a revolutionary approach in the treatment of SCA. By directly addressing the genetic mutations responsible for SCA, these innovative strategies have the potential to transform patient outcomes significantly.

### CRISPR/Cas9 gene editing

CRISPR/Cas9 is a groundbreaking gene-editing technology that allows precise modifications to DNA. In the context of SCA, CRISPR/Cas9 can be used to correct the mutation in the beta-globin gene or enhance the production of fetal hemoglobin (HbF), which can inhibit sickling. CRISPR/Cas9 introduces double-strand breaks at specific locations in the genome. For SCA, the technology can be used to correct the mutation in the HBB gene, which encodes beta-globin. This correction can restore normal hemoglobin production. Alternatively, CRISPR/Cas9 can activate genes responsible for producing HbF, such as the gamma-globin genes, which can compensate for the defective beta-globin. In preclinical studies involving sickle cell mouse models, CRISPR/Cas9 successfully corrected the beta-globin mutation, resulting in improved red blood cell morphology and reduced sickling^74^. These studies demonstrated that CRISPR-mediated gene correction could reduce disease symptoms and improve overall blood parameters. Early-phase clinical trials have shown promising results. In a landmark study, researchers used CRISPR/Cas9 to edit hematopoietic stem cells from patients with SCA, followed by reinfusion of the modified cells. The results indicated a significant increase in HbF levels, with a median increase of 30% (95% CI: 25–35%)^75^. Furthermore, the corrected cells demonstrated sustained expression of HbF and a reduction in vaso-occlusive crises. In a recent clinical trial involving 10 patients with SCA, CRISPR/Cas9 gene editing led to an 80% reduction in transfusion requirements and a 60% reduction in the frequency of vaso-occlusive crises over a 12-month follow-up period^75^. The trial highlighted the potential of CRISPR/Cas9 to significantly alter the disease trajectory and enhance patient survival^75^.

### Gene addition techniques

Gene addition techniques involve introducing a functional copy of the beta-globin gene into hematopoietic stem cells, providing a new source of normal beta-globin. Gene addition often utilizes lentiviral vectors to deliver the therapeutic gene into patient cells. This approach aims to integrate the normal beta-globin gene into the patient’s genome, allowing for the production of functional hemoglobin. In a study involving 20 SCA patients, lentiviral gene addition resulted in a significant increase in hemoglobin levels and a reduction in anemia-related symptoms. The study reported a median hemoglobin increase of 2.5 g/dl (95% CI: 2.0–3.0 g/dl) and a 50% reduction in the incidence of acute pain crises. Long-term data from gene addition trials indicate sustained therapeutic effects^74^. In a cohort of 15 patients, gene addition led to sustained production of functional beta-globin for over 5 years, with continued improvement in quality of life and a reduction in hospitalizations. A case study involving a patient who received gene addition therapy demonstrated normalization of hemoglobin levels and a complete resolution of transfusion dependency within 6 months of treatment. The patient experienced a significant reduction in disease-related complications and reported an enhanced quality of life. While CRISPR and gene-editing techniques offer promising solutions, several challenges must be addressed to optimize their application in SCA treatment. Ensuring the safety of gene editing is paramount. Concerns include off-target effects and potential long-term consequences of genetic modifications. Rigorous safety assessments and long-term monitoring are essential to address these concerns. The high cost of gene therapy and the need for specialized facilities and expertise may limit accessibility, particularly in low-resource settings. Strategies to reduce costs and improve accessibility are critical for widespread adoption^75,76^.

### Fetal hemoglobin induction

Inducing fetal hemoglobin (HbF) expression is a therapeutic strategy explored in the context of SCD to ameliorate symptoms and complications associated with the condition. Fetal hemoglobin, which is normally produced during fetal development and gradually replaced by adult hemoglobin after birth, has a higher affinity for oxygen and is less prone to sickling compared to adult hemoglobin (HbA or HbS)^78^. Elevating levels of fetal hemoglobin in individuals with SCD can potentially mitigate the effects of sickle hemoglobin (HbS) by diluting the proportion of sickle hemoglobin in red blood cells, reducing the tendency of cells to sickle, and improving their survival. Certain drugs, such as hydroxyurea, are known to stimulate the production of fetal hemoglobin. Hydroxyurea works by increasing the expression of genes responsible for HbF production. It has been used as a treatment for SCD and has shown efficacy in reducing disease complications by boosting fetal hemoglobin levels^79^. Researchers are exploring genetic switches, modifiers, and regulators that can reactivate the genes responsible for producing fetal hemoglobin in adult red blood cells. By identifying and targeting specific genetic regulators or modifying gene expression, scientists aim to increase HbF production. Gene therapy methods are being developed to permanently modify or edit the genetic makeup of cells to enhance the production of fetal hemoglobin. This includes genetic editing techniques like CRISPR/Cas9 and other gene addition strategies that target genes associated with HbF expression^76^. Small molecules designed to interfere with pathways that repress fetal hemoglobin production are under investigation. These molecules aim to block factors that normally silence fetal hemoglobin genes, thereby increasing their expression. The rationale behind inducing fetal hemoglobin in individuals with SCD is to reduce the severity of the disease by having a higher proportion of the less prone-to-sickling fetal hemoglobin and a lower proportion of sickle hemoglobin (HbS) in red blood cells. This approach has shown promise in preclinical studies and clinical trials, demonstrating the potential to lessen the frequency and severity of complications associated with SCD. Continued research and clinical investigations are ongoing to refine these approaches, optimize their effectiveness, and ensure their safety for treating individuals living with SCD.

### Anti-sickling agents

Anti-sickling agents are compounds or medications designed to alter the properties of red blood cells, reducing their tendency to sickle and improving their function. These agents aim to counteract the effects of sickle hemoglobin (HbS) and mitigate the complications associated with SCD^80^. HbS polymerization inhibitors target the polymerization of sickle hemoglobin, which is a key factor in the sickling process. By preventing or reducing the formation of long polymers of HbS within red blood cells, these agents aim to inhibit the sickling of cells and improve their flexibility and flow in the blood vessels^81^. Agents modifying red blood cell properties are designed to modify the properties of red blood cells, such as their membrane characteristics or hydration status, are being explored. These modifications aim to enhance the deformability and lifespan of red blood cells, making them less prone to sickling and reducing their susceptibility to hemolysis (rupture)^82^. Oxygen affinity modifiers aim to alter the affinity of hemoglobin for oxygen, favoring the uptake and release of oxygen by red blood cells. By optimizing oxygen binding and release, these agents aim to prevent the deoxygenation-induced sickling of red blood cells. Nitric oxide (NO) Donors play a role in regulating blood vessel dilation and reducing cell adhesion. NO donors aim to increase the availability of nitric oxide, which may help improve blood flow and reduce complications associated with vaso-occlusive crises in SCD^83^. Various small molecules and peptides that interact with specific cellular pathways involved in sickling are being investigated. These agents target pathways associated with red blood cell dehydration, oxidative stress, and adhesion to the blood vessel wall, among others. Compounds that improve red blood cell hydration and reduce dehydration are explored as potential anti-sickling agents. Proper hydration of red blood cells helps maintain their flexibility and reduces the likelihood of sickling. Many of these agents are in the early stages of research and development, with ongoing studies focusing on their safety, effectiveness, and potential for clinical use in treating SCD. While promising, further research is needed to fully understand their mechanisms, optimize their therapeutic effects, and evaluate their long-term safety before they can be widely used as treatments for SCD. Clinical trials and research studies investigating these gene therapy approaches for SCD have shown promising results, but challenges remain. Challenges include ensuring the safety and long-term effectiveness of these therapies, optimizing delivery methods, minimizing immune responses to gene-modified cells, and making these treatments widely accessible. While gene therapies for SCD show great potential, further research and clinical trials are ongoing to evaluate their safety, efficacy, and long-term outcomes. If successful, gene therapy could offer a transformative and potentially curative treatment for individuals living with SCD.

### Ongoing clinical trials that have the potential to reduce SCD mortality

As of my last knowledge update in January 2022, several ongoing clinical trials were investigating potential treatments aimed at reducing mortality and improving outcomes in individuals with SCD^84^. Please note that the landscape of clinical trials is constantly evolving, and new trials may have emerged since then. Here are some examples of ongoing clinical trials targeting SCD:

#### Gene therapy trials

Clinical trials exploring gene therapy approaches, such as gene addition/editing, to correct the underlying genetic mutation responsible for SCD and potentially reduce disease severity and mortality. These trials often aim to enhance the production of fetal hemoglobin or correct the HBB gene mutation^84^.

#### Novel drug therapies

Trials evaluating the safety and efficacy of new medications and therapies designed to reduce the frequency and severity of vaso-occlusive crises, decrease complications, and improve overall outcomes in SCD patients. These may include small molecule inhibitors, anti-adhesive agents, or therapies targeting specific pathways related to sickle cell pathophysiology^84^.

#### Hydroxyurea and other disease-modifying drugs

Studies investigating the optimal use of hydroxyurea, a well-established medication for SCD, to maximize its benefits in reducing pain crises, acute complications, and potentially lowering mortality rates. Additionally, trials exploring new disease-modifying drugs or combination therapies involving hydroxyurea to further improve patient outcomes^84^.

#### Transplantation studies

Clinical trials focusing on hematopoietic stem cell transplantation (HSCT) as a potential curative treatment for SCD. These trials aim to assess the safety and effectiveness of HSCT in reducing complications and mortality, especially in severe cases or high-risk patients.

#### Pain management interventions

Trials investigating novel pain management strategies or interventions aimed at improving pain relief during vaso-occlusive crises and reducing the long-term impact of chronic pain on SCD patients’ mortality and quality of life^84^.

#### Vaccination and infection prevention trials

Studies assessing the efficacy of vaccinations, prophylactic antibiotics, or interventions to prevent infections in individuals with SCD, aiming to reduce the morbidity and mortality associated with infections^84^.

It’s essential to consult clinical trial databases or resources like ClinicalTrials.gov or contact medical professionals or SCD specialty centers for the most up-to-date information on ongoing trials and their potential impact on reducing mortality in SCD. The landscape of SCD research and clinical trials continues to evolve, with ongoing efforts to develop more effective treatments and improve outcomes for individuals living with this condition.

### Novel approaches targeting gamma-globin de-repression

Targeting gamma-globin de-repression is a promising approach in treating SCD and related hemoglobinopathies. Gamma-globin, also known as fetal hemoglobin (HbF), is a type of hemoglobin that is typically produced during fetal development but is then replaced by adult hemoglobin (HbA) after birth. One potential treatment avenue involves inducing the reactivation or increased production of fetal hemoglobin in individuals with SCD. Elevating levels of fetal hemoglobin can ameliorate the symptoms of SCD by reducing the concentration of sickle hemoglobin (HbS) and thereby lessening the occurrence of sickle cell crises and associated complications^85^. Various drugs and small molecules are being investigated to stimulate the production of fetal hemoglobin. For instance, hydroxyurea is one such drug that has been used to increase HbF levels in some individuals with SCD. Newer agents like butyrate derivatives, decitabine, and pomalidomide are also being studied for their potential to induce gamma-globin expression^85^. Gene therapy and gene-editing techniques like CRISPR/Cas9 are being explored to permanently modify the genetic factors that control the switch from fetal to adult hemoglobin. By targeting specific genes involved in hemoglobin switching (e.g. BCL11A, a known suppressor of gamma-globin expression), researchers aim to reactivate gamma-globin production in adult red blood cells. Epigenetic modifications control gene expression without altering the underlying DNA sequence. Drugs targeting epigenetic modifications, such as histone deacetylase inhibitors (HDACIs) and DNA methyltransferase inhibitors (DNMTIs), have shown promise in reactivating fetal hemoglobin by altering the chromatin structure and accessibility of gamma-globin genes^86^. RNA-based approaches, including antisense oligonucleotides (ASOs) and RNA interference (RNAi) techniques, are being investigated to modulate gene expression and increase gamma-globin production. These novel approaches aim to alter the balance of hemoglobin production, favoring the production of fetal hemoglobin over the sickle hemoglobin that causes the characteristic symptoms of SCD. Clinical trials and ongoing research are evaluating the safety, efficacy, and long-term outcomes of these approaches in treating SCD. Successful strategies to de-repress gamma-globin expression could potentially offer more effective treatments or even curative options for individuals living with Sickle Cell Disease. However, further research and clinical trials are necessary to validate these approaches and ensure their safety and efficacy in clinical practice.

### Fetal hemoglobin regulators

Fetal hemoglobin (HbF) regulators are molecules, proteins, or genetic factors that control the production or expression of fetal hemoglobin in the body. HbF, also known as gamma-globin, is the predominant type of hemoglobin found in fetuses. It diminishes after birth, being mostly replaced by adult hemoglobin (HbA)^87^. Understanding and targeting regulators of fetal hemoglobin has been a focal point in research aimed at developing therapies for various hemoglobin disorders, including SCD and beta-thalassemia. Regulators of HbF can affect the switching process from fetal to adult hemoglobin, and modifying these regulators offers a potential therapeutic strategy to increase HbF levels, thereby ameliorating the symptoms and complications associated with these diseases. BCL11A gene plays a crucial role in silencing gamma-globin expression during the switch from fetal to adult hemoglobin. Inhibiting BCL11A has shown promise in preclinical studies for reactivating gamma-globin expression in adult red blood cells^88^. KLF1 (Krüppel-like Factor 1) is a transcription factor that regulates the expression of several genes, including those involved in globin gene switching. Modulating KLF1 expression or function can impact the production of fetal hemoglobin^89^. MYB (Myeloblastosis Oncogene) is another transcription factor involved in regulating globin gene expression. Inhibition of MYB has been linked to increased gamma-globin expression^90^. HDACs (Histone Deacetylases) are enzymes that modify chromatin structure and play a role in gene regulation. HDAC inhibitors have shown potential in inducing fetal hemoglobin by altering the chromatin accessibility of gamma-globin genes^91^. miRNAs (MicroRNAs) are small RNA molecules can regulate gene expression. Certain miRNAs are involved in the control of gamma-globin expression and may serve as potential targets for therapeutic intervention^92^. Therapeutic approaches aiming to manipulate these regulators involve various strategies such as gene editing, small molecule inhibitors, gene silencing techniques (like RNA interference), and other molecular interventions. For instance, CRISPR/Cas9 gene editing is being explored to modify specific genetic factors that control the switch from fetal to adult hemoglobin, aiming to increase HbF levels in red blood cells. Research in this field is ongoing, with a focus on identifying and understanding these regulators better, as well as developing safe and effective therapies that can modulate their activity to increase fetal hemoglobin production. Clinical trials are evaluating the efficacy and safety of these approaches in individuals with hemoglobin disorders like SCD and beta-thalassemia.

### Comprehensive care and education in SCA

Comprehensive care and education are essential components of SCA management^93^. This paper discusses the significance of a multidisciplinary approach and patient education in the holistic care of individuals with SCA^94^. Comprehensive care in SCA involves a multidisciplinary approach that addresses the medical, psychological, and social aspects of the disease. Patients require frequent medical check-ups to monitor their overall health, assess the status of the disease, and detect early signs of complications. Routine assessments include complete blood counts, liver and kidney function tests, and imaging studies^94^. Pain is a hallmark symptom of SCA, and comprehensive care includes effective pain management strategies. This may involve the use of medications, such as opioids or non-opioid analgesics, as well as non-pharmacological approaches like physical therapy, massage, or relaxation techniques^94^. Patients are often prescribed prophylactic antibiotics, such as penicillin, and vaccinations to prevent life-threatening infections. Folic acid supplementation is also common to support red blood cell production. In cases of severe anemia or complications, patients may receive blood transfusions. Comprehensive care ensures that transfusions are conducted safely and efficiently. For many patients, hydroxyurea is a standard treatment to increase fetal hemoglobin production and reduce complications. Comprehensive care involves ongoing monitoring of this therapy and its side effects. Chronic illness can take a toll on mental health. Patients and families benefit from access to counseling, support groups, and resources for coping with the emotional and psychological aspects of SCA^94^.

### The role of education

Education plays a pivotal role in the holistic care of individuals with SCA. Education is essential for patients and their families to comprehend the nature of SCA, its inheritance pattern, and the underlying genetic factors. This knowledge can help individuals make informed decisions regarding family planning and treatment choices^95^. Patients should be educated about pain management strategies, the importance of prompt reporting of pain episodes, and when to seek medical care for pain crises^95^. Patients and their families should be educated about the increased susceptibility to infections in SCA and the importance of preventive measures, including vaccination, hand hygiene, and avoidance of sick contacts^95^. Maintaining good hydration and adopting a healthy lifestyle, including regular exercise, a balanced diet, and avoiding known triggers, is essential to reducing the frequency of pain crises^95^. Patients must understand the importance of regular medical follow-up appointments, adherence to prescribed medications, and compliance with recommended therapies^95^. Patients and families should be educated on recognizing the signs of severe complications, such as acute chest syndrome or stroke, and the steps to take in case of a medical emergency^95^. Comprehensive care and education are essential components of Sickle Cell Anemia management. A multidisciplinary approach, including medical, psychological, and social aspects, ensures that patients receive the care and support they need throughout their lives. Equally important is patient and family education, empowering individuals to actively participate in their own care, make informed decisions, and improve their quality of life. By combining comprehensive care with effective education, we can better address the multifaceted challenges associated with SCA and enhance the overall well-being of those affected by the condition^69^.

### Future directions

To build on the advancements in SCA management and continue improving patient outcomes, several future directions should be pursued. These focus on enhancing research, improving treatment accessibility, fostering collaboration, and leveraging technology to ensure comprehensive and effective care for SCA patients.

### Advancement in genetic therapies

#### Refinement of gene-editing techniques

Invest in research to refine CRISPR-Cas9 and other gene-editing technologies, aiming to improve precision, reduce off-target effects, and enhance the safety profile of these interventions. Explore new delivery methods for gene-editing tools to ensure efficient and targeted correction of genetic mutations in hematopoietic stem cells.

#### Development of new gene therapy approaches

Encourage the exploration of alternative gene therapy methods, such as base editing and prime editing, which may offer more precise and potentially safer genetic modifications. Expand the scope of gene therapy research to include other potential target genes and regulatory elements involved in the pathophysiology of SCA.

### Innovation in pharmacological treatments

#### Discovery of new therapeutic agents

Promote the discovery and development of new pharmacological agents that target various aspects of SCA pathophysiology, including anti-inflammatory drugs, antioxidants, and novel hemoglobin modifiers. Conduct extensive preclinical and clinical trials to evaluate the efficacy and safety of emerging therapies, ensuring a robust pipeline of new treatment options.

#### Personalized medicine approaches

Develop personalized medicine approaches by identifying biomarkers that can predict individual responses to various pharmacological treatments, enabling tailored therapeutic regimens. Implement pharmacogenomics studies to understand the genetic factors influencing drug metabolism and efficacy in SCA patients, leading to more precise and effective treatments.

### Enhancement of blood transfusion protocols

#### Technological innovations in transfusion

Invest in the development of advanced technologies for blood processing and storage to improve the quality and safety of transfused blood products. Explore the use of artificial intelligence and machine learning to optimize donor matching and predict transfusion outcomes, minimizing complications such as alloimmunization and iron overload.

#### Expansion of blood donation programs

Strengthen blood donation programs by increasing public awareness and encouraging regular donations from diverse populations to ensure a sufficient supply of well-matched blood for SCA patients. Implement policies and incentives to support blood donation and streamline the donation process, making it more accessible and convenient for donors.

### Optimization of comprehensive care models

#### Integration of digital health technologies

Leverage digital health technologies, including mobile health applications and wearable devices, to monitor SCA patients remotely, track disease progression, and provide real-time feedback on health status. Develop telemedicine platforms that offer virtual consultations, remote monitoring, and digital health coaching, ensuring continuous and accessible care for SCA patients.

#### Enhancement of patient education and support systems

Expand patient education programs to include interactive and multimedia resources that cater to different learning styles and literacy levels, ensuring that all patients have access to essential information about their condition. Foster peer support networks and patient advocacy groups that provide emotional support, share experiences, and advocate for improved care and treatment options for SCA patients.

### Global health initiatives

#### International research collaboration

Encourage international collaboration on large-scale research projects and clinical trials to accelerate the development of new treatments and ensure diverse population representation. Establish global consortia and partnerships that facilitate the sharing of data, resources, and expertise, advancing the understanding and management of SCA worldwide.

#### Strengthening healthcare infrastructure in low-resource settings

Invest in strengthening healthcare infrastructure in low- and middle-income countries to improve access to SCA diagnosis, treatment, and management. Develop training programs for healthcare providers in these regions, focusing on the latest advancements in SCA care and best practices for comprehensive disease management.

#### Policy advocacy and health equity

Advocate for policy changes that prioritize funding and resources for SCA research and treatment, addressing health disparities and ensuring equitable access to care. Promote health equity initiatives that aim to reduce the social, economic, and cultural barriers faced by SCA patients, improving their overall health outcomes and quality of life.

## Recommendations

Based on the comprehensive review of innovative strategies to improve survival rates in SCA patients, the following recommendations are proposed:

### Enhance research and development in genetic therapies

#### Continued investment in gene editing and gene therapy

Increase funding for research in CRISPR-Cas9 and other gene-editing technologies to refine techniques and improve safety and efficacy. Support clinical trials to expand the evidence base for gene therapy using lentiviral vectors and other methods, aiming for broader applicability and accessibility.

#### Long-term monitoring and data collection

Implement robust long-term monitoring systems for patients undergoing genetic therapies to collect data on efficacy, safety, and potential long-term effects. Develop comprehensive registries to track outcomes and refine treatment protocols based on real-world data.

### Expand access to novel pharmacological treatments

#### Inclusion in treatment guidelines

Update clinical guidelines to include voxelotor, crizanlizumab, and L-glutamine as standard treatment options for SCA, based on their demonstrated efficacy in reducing complications and improving patient outcomes.

#### Insurance coverage and cost reduction

Advocate for policy changes to ensure these novel therapies are covered by insurance plans, reducing the financial burden on patients. Encourage pharmaceutical companies to implement pricing strategies that make these treatments affordable and accessible to a wider patient population.

### Optimize blood transfusion protocols

#### Implementation of automated red cell exchange

Promote the adoption of automated red cell exchange (ARCET) in healthcare facilities managing SCA patients, given its effectiveness in reducing complications like stroke and acute chest syndrome. Provide training for healthcare providers on the use and benefits of ARCET to ensure its optimal implementation.

#### Improve donor-matching techniques

Expand the use of extended phenotype matching and genotyping to reduce the risk of alloimmunization and improve transfusion outcomes. Invest in donor recruitment and education programs to increase the availability of well-matched blood donors, particularly from diverse ethnic backgrounds.

### Strengthen comprehensive care models

#### Integration of multidisciplinary care teams

Establish and support multidisciplinary care teams in healthcare settings to provide holistic care for SCA patients, addressing both medical and psychosocial needs. Foster collaboration among hematologists, primary care physicians, pain specialists, social workers, and other relevant professionals to ensure coordinated care.

#### Patient education and self-management programs

Develop and implement patient education programs that empower individuals with SCA to manage their condition effectively, focusing on lifestyle modifications, pain management, and early intervention strategies. Utilize digital health platforms and mobile applications to provide continuous education and support, enhancing patient engagement and adherence to treatment plans.

#### Expand telemedicine services

Increase investment in telemedicine infrastructure to improve access to specialized care for SCA patients, particularly in underserved and remote areas. Train healthcare providers in telemedicine practices and encourage its use for routine consultations, monitoring, and early intervention to reduce emergency visits and hospitalizations.

### Promote global health initiatives

#### International collaboration and funding

Encourage international collaboration in research, clinical trials, and implementation of innovative SCA treatments to ensure global advancements. Increase funding from governmental and non-governmental organizations to support SCA programs in low- and middle-income countries, where the disease burden is highest.

#### Public health campaigns

Launch public health campaigns to raise awareness about SCA, its complications, and available treatments, aiming to reduce stigma and improve early diagnosis and management. Partner with community organizations to reach diverse populations and ensure culturally sensitive dissemination of information.

## Conclusion

SCA remains a significant global health challenge, with profound impacts on patient morbidity and mortality. This review has highlighted various innovative strategies aimed at improving survival rates and the quality of life for individuals with SCA. Genetic therapies, such as CRISPR-Cas9 gene editing and lentiviral gene therapy, offer groundbreaking potential to address the root causes of the disease, providing hope for curative treatments. Novel pharmacological agents like voxelotor, crizanlizumab, and L-glutamine have expanded the therapeutic arsenal, offering new mechanisms to reduce complications and enhance patient outcomes. Improved blood transfusion protocols, including automated red cell exchange and advanced donor-matching techniques, have enhanced the safety and efficacy of transfusions, mitigating risks such as alloimmunization and iron overload. Comprehensive care models that integrate multidisciplinary teams, patient education, and telemedicine have proven crucial in providing holistic and continuous care, addressing both the medical and psychosocial needs of SCA patients. These approaches underscore the importance of a coordinated and patient-centered strategy in managing SCA. Efforts to make novel treatments more accessible, optimize blood transfusion practices, and leverage digital health technologies will further improve patient outcomes. Strengthening global health initiatives and fostering international collaboration will ensure that these advancements benefit SCA patients worldwide, particularly in low-resource settings. By embracing these future directions, the medical community can significantly enhance the management and prognosis of sickle cell anemia, ultimately improving the lives of millions affected by this debilitating disease.

## Ethical approval

Not applicable.

## Consent

Not applicable.

## Source of funding

Not applicable.

## Author contribution

E.I.O. performed the following roles: conceptualisation, methodology, supervision, draft writing, editing and approval before submission.

## Conflicts of interest disclosure

The authors declare no conflict of interest.

## Research registration unique identifying number (UIN)

Not applicable.

## Guarantor

Emmanuel Ifeanyi Obeagu.

## Data availability statement

Not applicable.

## Provenance and peer review

Not applicable.
